# Generating aptamers towards human sperm cells using massively parallel sequencing

**DOI:** 10.1007/s00216-021-03562-7

**Published:** 2021-08-05

**Authors:** James Gooch, Sireethorn Tungsirisurp, Hayley Costanzo, Richard Napier, Nunzianda Frascione

**Affiliations:** 1 Department of Analytical, Environmental & Forensic Sciences, King’s College London, London, SE1 9NH UK; 2 School of Life Sciences, The University of Warwick, Coventry, CV4 7AL UK

**Keywords:** Aptamers, Sperm cells, SELEX, Massively parallel sequencing, Next-generation sequencing, Microscale thermophoresis

## Abstract

Determining the presence of sperm cells on an item or swab is often a crucial component of sexual offence investigation. However, traditional histological staining techniques used for the morphological identification of spermatozoa lack both specificity and sensitivity, making analysis a complex and time-consuming process. New methods for the detection of sperm cells based on aptamer recognition may be able to overcome these issues. In this work, we present the selection of ssDNA aptamers against human sperm cells using Cell-SELEX and massively parallel sequencing technologies. A total of 14 rounds of selection were performed following a modified Cell-SELEX protocol, which included additional steps for the isolation of spermatozoa from seminal fluid. Massively parallel sequencing using the Illumina Miseq platform was conducted on enriched aptamer pools to elucidate the structure of potential binders. A custom bioinformatics pipeline was also developed using Galaxy for the automated processing of sequencing datasets. This data revealed several promising aptamer candidates, which were shown to selectively bind sperm cells through both microscale thermophoresis and enzyme-linked oligonucleotide assays. These aptamers have the potential to increase the efficiency of sexual offence casework by facilitating sperm detection.

**Graphical abstract:**

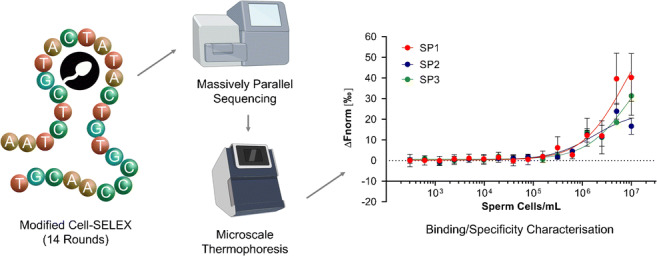

## Introduction

Despite their increasing frequency, conviction rates for sexual offences remain much lower than other violent crimes. In fact, only 1.5% of rapes reported to the police result in the conviction of a perpetrator [1]. Approximately half of these reported cases fail to progress past the investigative stage of the criminal justice process due to ‘evidence issues’ [2]. The effective detection of forensic evidence has therefore become of paramount importance in sexual offence investigation as the primary method of linking an individual to a crime or exonerating those that have been wrongly accused.

One of the most valuable forms of forensic evidence in cases of male-perpetrated sexual assault is the presence of seminal fluid, either in the form of stains left behind on items or on swabs taken from victims during a forensic medical examination [3]. Methods used by forensic laboratories to confirm the presence of semen are currently based on the microscopic examination of sperm cells extracted from suspected fluid samples [4]. This examination is often facilitated by the use of histological staining techniques designed to enhance sperm cell visualization, allowing for easier assessment of cell size, number, and morphology [5]. Despite routine use in forensic laboratories for over 40 years, stains such as haematoxylin-eosin, Christmas tree, and alkaline fuchsin are known to lack both specificity and sensitivity, making the identification of sperm samples extremely challenging (especially if they are degraded, mixed, or contaminated) [6].

In recent years, several new assays for sperm cell detection based on antibody recognition have appeared on the market in an attempt to overcome these limitations. These assays, developed as part of the Sperm Hy-Liter product range, use anti-sperm monoclonal antibodies functionalised with Alexa 488 fluorophores to label sperm cells, allowing them to be visualized on glass slides using standard fluorescence microscopy [7]. However, despite initial promise, these techniques have yet to be widely adopted by the forensic community due to the high cost associated with antibody-based testing reagents [8].

Emerging as a synthetic alternative to antibodies, aptamers are short sequences of ssDNA or RNA that can be raised in vitro to bind to virtually any given target [9]. Using the Systematic Evolution of Ligands by Exponential Enrichment (SELEX) process, aptamers can be selected against small molecules, large proteins, or whole cells through the cyclical target-incubation and enrichment of randomized oligonucleotide libraries [10]. Whilst analogous to antibodies in terms of binding affinity, aptamers possess several advantages over their protein counterparts, including enhanced stability [11], greater speed of production [12], and ease of chemical modification [13]. Importantly, aptamers can also be mass-produced at low cost with no batch-to-batch variation [14]. Given such advantages, we hypothesised that nucleic acid aptamers may be a suitable substitute to antibodies for use in sperm cell detection assays [15].

One attempt at producing oligonucleotide ligands towards human sperm cells has already been undertaken by Katilius et al. on behalf of Somalogic, Inc., a protein biomarker discovery and diagnostics company, using a proprietary enrichment method [16]. This study saw the development of ‘SOMAmer’ affinity reagents, ssDNA molecules modified to include functional groups that mimic amino acid side chains, for the purpose of capturing and separating sperm cells from vaginal epithelial cells in forensic casework (rather than for detection purposes). However, the sequence structures of these reagents have yet to be disclosed. Another study by Farini et al. also reported the development of aptamers specific to heat-damaged bovine spermatozoa via the Cell-SELEX process, demonstrating the suitability of this method for generating ligands towards whole sperm cell targets [17].

Efforts were therefore made towards the selection of ssDNA aptamers against human sperm cells following a modified version of a popular Cell-SELEX protocol previously reported by Sefah et al. [18]. This protocol included the use of additional steps for the isolation of spermatozoa from whole seminal fluid (rather than preparing cells through culturing techniques), as well as the identification of potential binding sequences by massively parallel sequencing (MPS, also known as ‘next-generation sequencing’ and ‘high-throughput sequencing’). MPS was chosen for the analysis of enriched aptamer pools in order to increase the chances of identifying binding candidates at a much earlier stage of selection, provide a greater level of sequence information from pooled samples, and avoid the time-consuming cloning and colony-picking processes associated with conventional Sanger sequencing. A bespoke bioinformatics pipeline was also developed for the processing of these MPS datasets using the web-based genomics toolset ‘Galaxy’ [19]. Whilst specifically constructed for use within this study, this pipeline may find extended use for the handling of MPS data obtained from any future SELEX experiment employing a randomized N40 ssDNA library (or a library of any length given minor workflow alterations). Aptamer candidates identified using this pipeline were lastly tested for their ability to selectively bind sperm cells over negative controls using two bimolecular interaction analysis techniques, Microscale Thermophoresis (MST) and enzyme-linked oligonucleotide assay (ELONA). To the best of our knowledge, this study represents the very first use of MST to characterize the binding of aptamers to any whole-cell target. Such aptamers may have the ability to streamline forensic casework practices by allowing the detection of human sperm cells on objects or swabs with a higher degree of specificity and sensitivity compared to traditional histological staining methods and at a lower cost than commercial antibody-based assays.

## Materials and methods

### Reagents

For sperm cell isolation, Percoll was obtained from Sigma-Aldrich (Dorset, UK). Human tubal fluid (HTF) was prepared as a ×10 concentrate through the addition of 29.655 g NaCl, 1.75 g KCl, 250 mg MgSO_4_·7H_2_O, 250 mg KH_2_PO_4_, 2.5 g glucose, 2.6 g HEPES, 1 g NaHCO_3_, 1.5 g CaCl_2_·2H_2_O, and 3 g human serum albumin (HSA) to 350 mL sterile distilled water (SDW). This solution was then adjusted to pH 7.4 and made up to a total volume of 500 mL using SDW. Dilution of this concentrate in SDW was used to prepare all ×1 HTF solutions.

For cell incubation and elution, an ssDNA library containing two fixed regions, each 18 nucleotides in length, flanking randomized N40 base sequences, (5′-ATCCAGAGTGACGCAGCA-N40-TGGACACGGTGGCTTAGT-3′) was obtained (HPLC purified) from Integrated DNA technologies (IA, USA). Aptamer binding buffer was prepared through the addition of 2.25 g glucose, 500 mg BSA, and 238 mg MgCl_2_ to 500 mL of ×1 phosphate-buffered saline (PBS).

All PCR reaction components, including ×10 PCR buffer, 50 mM MgCl_2_ solution, 10 mM dNTP mixture, and Platinum Taq DNA polymerase were obtained from Thermo Fisher Scientific (CA, USA). Fluorescently labelled forward (5′-6-FAM-ATCCAGAGTGACGCAGCA-3′) and biotin-functionalised reverse (5′-biotin-ACTAAGCCACCGTGTCCA-3′) primers were both obtained (HPLC purified) from Integrated DNA technologies (IA, USA). Non-labelled versions of both forward and reverse sequences were also procured.

For gel electrophoresis, agarose was obtained from Sigma-Aldrich (Dorset, UK). Tris-Acetate-EDTA (TAE) buffer (×50) and GelRed nucleic acid gel stain (×10,000 in H_2_O) were both purchased from Cambridge Bioscience (Cambridge, UK), whilst HyperLadder 25 bp (also containing ×6 loading dye) was obtained from Bioline (Toronto, Canada).

For the separation of double-stranded DNA (dsDNA), Pierce NeutrAvidin agarose resin was purchased from Thermo Fisher Scientific (CA, USA). Corning Costar Spin-X and Amicon Ultra-0.5 mL (3 kDa MWCO) filter units were both obtained from Sigma-Aldrich (Dorset, UK).

All DNA sequencing reagents, including the NEBNext Ultra DNA Library Prep Kit for Illumina, NEBNext Multiplex Oligos for Illumina (both New England Biolabs, MA, USA), and Agencourt AMPure XP Beads (Beckman Coulter, CA, USA), were provided by Dr. David Ballard from DNA Analysis at King’s, King’s College London.

For ELONA, Nunc MaxiSorp™ flat-bottom 96-well plates, 3,3′,5′-tetramethylbenzidine (TMB) substrate solutio,; 1 M sulphuric acid solution, and streptavidin-conjugated horseradish peroxidase (SA-HRP) were all obtained from Thermo Fisher Scientific (CA, USA).

### Body fluid collection

Seminal fluid and saliva were obtained upon informed consent from healthy donors and stored at 4 °C until the point of analysis. Blood samples were drawn by venipuncture upon informed consent from the same healthy donor and stored in a BD Vacutainer Plus tube (Oxford, UK) containing 3.2% sodium citrate coagulation preservative and stored at 4 °C until analysis.

Ethical clearance for the collection and use of body fluids for this study was granted by the King’s College London Biomedical Sciences, Dentistry, Medicine and Natural & Mathematical Sciences Research Ethics Subcommittee (reference number HR-17/18-5057). All experiments were performed in accordance with the Human Tissue Act 2004.

### Sperm cell isolation

Isolation of sperm cells from whole seminal fluid was performed using density gradient centrifugation methods outlined by the World Health Organization (WHO) [20]. First, an isotonic gradient was prepared through the addition of 1 mL of ×10 HTF to 9 mL of Percoll. This gradient was separated into two fractions which were then diluted in ×1 HTF to form 80% and 40% (v/v) Percoll suspensions. A 2 mL volume of the 40% suspension was then layered over the top of 2 mL of the 80% suspension in a 15-mL tube to create a discontinuous density gradient. Seminal fluid (1 mL) was then added to the top of the gradient and centrifuged for 20 min at 500 g. The ensuing supernatant was removed from the pelleted cells, which were then re-suspended and washed twice with 5 mL of ×1 HTF. Pelleted sperm cells were lastly re-suspended in 2 mL of SDW.

Cell concentrations were determined using a Countess II automated cell counter (Thermo Fisher Scientific, CA, USA) after mixing 10 μL of suspended cells with 10 μL of trypan blue solution.

### Cell-SELEX

#### Incubation and elution

For the first round of the Cell-SELEX process, 100 μL of 100 μM (10 nmol) N40 ssDNA library was added to 270 μL of aptamer binding buffer, heated to 95 °C for 5 min, and allowed to cool to room temperature. During this period, a volume of sperm cell suspension (obtained from the same single donor) yielding a total of 1 × 10^7^ cells was transferred into a 15-mL tube and centrifuged at 9000 *g* for 3 min. The resulting supernatant was then removed before the re-suspension of pelleted cells in 330 μL of aptamer binding buffer. These cells were then added to the 370 μL of cooled ssDNA library previously prepared and incubated for 1 h at 4 °C on a rotary shaker.

After incubation, this mixture was centrifuged at 9000 *g* for 3 min in order to pellet cells and bound ssDNA sequences. The resulting supernatant (comprised of remaining unbound sequences was removed before cells were re-suspended and washed (×3) with 1 mL of aptamer binding buffer. Bound ssDNA sequences were then eluted from cells through the addition of 500 μL DNase-free SDW with heating at 95 °C for 10 min. A final centrifugation step at 9000 *g* for 10 min was then used to pellet cells so that eluted sequences could be collected in the ensuing supernatant.

In all subsequent selection rounds (2–14), incubation was achieved through the addition of 200 μL ssDNA, diluted to a concentration of 1 μM in aptamer binding buffer, to cells also suspended in 200 μL of binding buffer. The total number of cells used for incubation was reduced by one million each cycle between SELEX rounds 2–5 (from 5 × 10^6^ to 1 × 10^6^ cells), in order to increase the stringency of the selection process. A total of 1 × 10^6^ cells were used in all further incubations (from rounds 6–14). Elution of sequences from cells in rounds 2 to 14 was also conducted using 600 μL of binding buffer, rather than 500 μL of SDW.

#### PCR amplification

In total, three different types of PCR amplification were performed throughout the SELEX process: (1) pre-PCR, (2) trial PCR, and (3) preparative PCR. However, the former of these was only carried out during the first round of selection.

After the first incubation of N40 ssDNA library with cells, pre-PCR amplification of the entire eluted pool was conducted to create multiple representative copies of all possible bound sequences, thereby avoiding the potential loss of aptamers during subsequent pipetting processes. This was achieved through the addition of 500 μL eluted sequences to 500 μL of a pre-PCR mix (Table 1), which was then run as 20 separate 50 μL reactions for 9 cycles on a Perkin-Elmer 9700 thermal cycler (Cambridge, UK) using the temperature programme found in Table 2.

**Table 1:** Reaction mixtures used for pre, trial, and preparative PCR during the SELEX process

Reagents	Pre-PCR (μL)	Trial PCR (μL)	Prep PCR (μL)
×10 PCR buffer	100.00	35.00	100.00
dNTP mixture (10 mM)	20.00	7.00	20.00
MgCl_2_ (50 mM)	40.00	14.00	40.00
Forward primer (100 μM)	5.00	1.75	5.00
Reverse primer (100 μM)	5.00	1.75	5.00
DNase-free SDW	327.00	254.45	727.00
Platinum *Taq* polymerase	3.00	1.05	3.00
Eluted ssDNA Pool	500.00	-	100.00

**Table 2:** Thermal cycling conditions used for pre, trial, and preparative amplification reactions

Stage	Temperature (°C)	Time (s)
Denature	95.0	120
Denature*	95.0	30
Annealing*	56.3	30
Extension*	72.0	30
Extension	92.0	180
Hold	4.0	∞

*Cycling stage

Trial PCR reactions of ssDNA eluted during each round of selection were carried out in order to determine the optimum number of amplification cycles required for subsequent preparative PCRs. This was conducted through the preparation of a trial PCR reaction mix, which was then split into six individual 45 μL volumes. To each of these volumes, 5 μL of eluted ssDNA was added, with the exception of one sample, to which SDW was instead used in order to act as a negative control. All samples were run using the same thermal cycling methods and temperature programme previously outlined, with one tube containing eluted ssDNA removed after 6, 8, 10, 12, and 14 cycles. Negative control samples were run for a maximum number of 14 cycles. Amplification products were then loaded alongside a HyperLadder 25-bp size determination marker onto a 4% agarose gel (made in 1× TAE and containing 0.01% GelRed), which was then run in 1× TAE buffer at 80 V for 100 min.

In order to generate the amount of ssDNA required for input into further selection rounds, 100 μL of ssDNA eluted from each incubation was lastly amplified after addition to a preparative PCR mix using the optimum number of cycles established during trial PCRs. Samples were run in 20 separate 50-μL reactions using the thermal cycling methods and temperature programme previously described. Agarose gel electrophoresis was used to confirm successful amplification in the same manner as trial PCR studies.

#### Strand separation

With dsDNA generated as a result of the amplification process, strand separation of PCR products from each round of selection was conducted via the use of an avidin-labelled resin to capture biotin-functionalised antisense strands. First, 200 μL Pierce NeutrAvidin agarose resin was added to two Corning Costar Spin-X filter units, which were then centrifuged at 500 *g* for 1 min in order to remove storage buffer. Resin beads were then preconditioned for 30 min using 500 μL of 200 mM NaOH to remove any labile avidin molecules, before further centrifugation under the same conditions. Resins were then washed (×5) with 500 μL of ×1 PBS with further centrifugation. Incubation of resin beads with 500 μL of preparative PCR products from each round of selection was then conducted for 30 min on an orbital shaker to bind dsDNA through biotin-avidin interaction. After this incubation, resins were centrifuged and washed (×5) with PBS in the same manner previously described. 500 μL of 200 mM NaOH was then added to the NeutrAvidin beads in order to separate fluorescently labelled sense strands, which were then collected after centrifugation at 1000 *g* for 10 min.

To remove NaOH, obtained ssDNA was placed into two Amicon Ultra-0.5 mL (3 kDa MWCO) filter units and centrifuged at 14,000 *g* for 30 min. DNase-free H_2_O (450 μL) was then added to the filters, which were then subjected to further centrifugation under the same conditions. De-salted ssDNA was finally collected by placing the filter units upside down in clean microfuge tubes and centrifuging at 1000 *g* for 7 min. The concentration of ssDNA was lastly quantified using a NanoDrop ND-1000 Spectrophotometer (Thermo Fisher Scientific, CA, USA).

### Massively parallel sequencing and data analysis

Enriched aptamer pools obtained from rounds 1, 2, 3, 8, 9, 10, and 14 of selection were subjected to MPS to elucidate the structure of potential binding sequences. First, dsDNA products were prepared from each pool by PCR amplification using the same reaction mix (with the exception of non-labelled primer sets), number of cycles, and temperature programme previously employed during the preparative PCRs for each respective round. Amplified products were then quantified using a NanoDrop ND-1000 Spectrophotometer (Thermo Fisher Scientific, CA, USA) before being diluted in SDW to a concentration of 2 ng/μL.

Samples were then prepared for sequencing using the NEBNext Ultra DNA Library Prep Kit for Illumina. Briefly, 1.4 μL End Prep Enzyme Mix and 2.9 μL End Repair Reaction Buffer (×10) were added to 25 μL of each dsDNA sample, which were then incubated for 30 min at room temperature and 30 min at 65 °C. Adaptors were then ligated to the ends of dsDNA sequences through the addition of 7.5 μL Blunt/TA Ligase Master Mix, 1.25 μL NEBNext adaptor for Illumina and 0.5 μL Ligation Enhancer. This mixture was then incubated at room temperature for 15 min before the addition of 2 μL USER Enzyme, with further incubation at 37 °C for 15 min. A post-ligation cleanup was then conducted using AMPure XP Beads with sequences eluted in 10 μL of 10 mM Tris-HCl, pH 8.5. A further PCR amplification of sequences was performed through the addition of 7.5 μL adaptor-ligated dsDNA to 12.5 μL NEBNext Ultra II Q5 Master Mix, 2.5 μL index primer, and 2.5 μL Universal PCR primer. This mixture was run for 8 cycles in a thermal cycler using the temperature programme found in Table 3. A final cleanup procedure was conducted using AMPure XP Beads before elution in ×1 TE buffer. All prepared samples were loaded onto a V2 reagent cartridge and sequenced using the Illumina Miseq platform (CA, USA).

**Table 3:** Thermal cycling temperature programme used for MPS sample preparation

Stage	Temperature (°C)	Time (s)
Denature	98.0	30
Denature*	98.0	10
Annealing*	65.0	75
Extension*	65.0	300
Hold	4.0	∞

*Cycling stage

Data analysis of raw sequence reads was performed using Galaxy, an open-source bioinformatics platform, following a custom-developed pipeline adapted from previous work conducted by Thiel et al. [21].

### Microscale Thermophoresis

Aptamer sequences SP1 (5′-CACTCTCACCTTCCTGTCACTCCTTTTTTCACTCTCACTC-3′), SP2 (5′-CACTCACTCTTTCCCTTATCTGCTCACTCTTCATTCACTC-3′), and SP3 (5′-CACCGTCCTTTTCTAGCATCTCTGTCACTCTTTCTCACTC-3′) labelled with 5′ 6-FAM fluorophores were synthesized by Integrated DNA Technologies (IA, USA) and prepared to an initial stock concentration of 40 nM in PBS supplemented with 0.05% Tween-20. Prior to use, all aptamer solutions were heated to a temperature of 95 °C for 5 min and snap-cooled on ice. A target dilution series was prepared by titrating sperm cells suspended in SELEX buffer in 16 steps 1:1 from an initial concentration of 2 × 10^7^/mL. For the measurement of binding affinity, 5 μL of each fluorescently labelled aptamer solution (40 nM) was mixed with 5 μL of all sperm cell dilutions to allow binding reactions to occur (with each sample prepared in triplicate). Reaction mixtures were drawn up using standard MST capillaries and loaded onto a Monolith NT.115 system (NanoTemper Technologies GmbH, Germany) at an ambient temperature of 25 °C. Instrument parameters were adjusted to 25% LED power and medium MST power for all samples. Identical experiments were also performed using dilutions of human red blood and salivary epithelial cells to assess the potential for non-specific aptamer binding.

Data obtained from all sample replicates were analysed by MO.Affinity Analysis software version 2.2.4, (NanoTemper Technologies GmbH, Germany) using the signal from an MST-on time of 15 s. Normalized fluorescence responses were transformed to ΔFnorm (‰) values (by subtracting baseline Fnorm values from all data points on the same curve) in order to correct for baseline differences and allow manual plotting against cell concentrations in a dose-response curve using GraphPad Prism software version 8.4.1 (GraphPad, CA, USA).

### Enzyme-linked oligonucleotide assay

Aptamer sequences SP1, SP2, and SP3, as well as a randomized control sequence (5′-ACTAAGCCACCGTGTCCA-3′), were synthesized with 5′ biotin groups by Integrated DNA Technologies (IA, USA) and prepared to an initial stock concentration of 1 μM in PBS supplemented with 5 mM MgCl_2_, 0.05% Tween-20 and 1% BSA. Prior to use, all aptamer solutions were heated to a temperature of 95 °C for 5 min and snap-cooled on ice. A series of sperm cell dilutions (1:10, 1:100, 1:200, 1:400, 1:800, and 1:1000 in carbonate/bicarbonate buffer pH 9.6, starting from an initial stock concentration of 4.7 × 10^6^ cells/mL) were prepared from isolated sperm samples obtained from three healthy donors. Each sperm dilution sample was then added in a 100 μL volume to a Nunc MaxiSorp flat-bottom 96-microwell plate (Thermo Scientific, CA, USA) for 1 h at 37 °C to coat the sample wells. After incubation, each plate was drained and all wells were washed three times with ELONA wash buffer (PBS, 5 mM MgCl_2_, Glucose and 0.05% Tween-20). Next, remaining well surfaces were blocked by incubation with 200 μL ELONA blocking buffer (PBS, 5 mM MgCl_2_ and 1% BSA) for 1 h at room temperature on a rotary shaker. All wells were again drained and washed three times with ELONA buffer before the addition of 1 μM aptamer (or control sequence) with incubation for 1 h on a rotary shaker. Plates were drained and washed for a third time using ELONA wash buffer to remove any unbound aptamers prior to the addition of 100 μL of 1 μg/mL SA-HRP, with plates left for 45 min at room temperature on a rotary shaker. A final drain and wash with ELONA wash buffer was lastly performed on each plate before the addition of 100 μL TMB substrate for 20 min at room temperature with shaking. TMB substrate reactions were paused using 50 μL 1 M sulphuric acid solution. Measurements of substrate absorbance were performed using an Opsys MR UV-Vis microplate reader (Dynex Technologies, VA, USA) at 450 nm.

## Results and discussion

### Aptamer selection

**Fig. 1:**
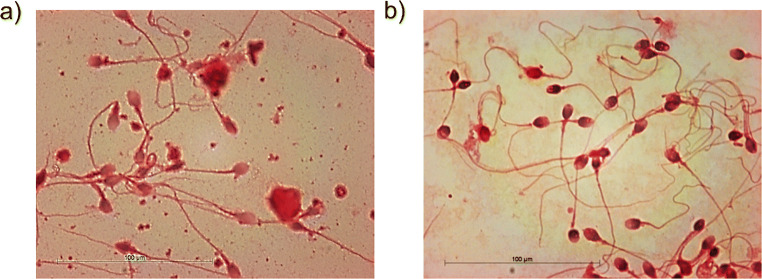
Microscopic examination of haemotoxylin and eosin-stained sperm cells. **a** Pre- and **b** post-isolation using a density gradient centrifugation procedure

Aptamers were selected according to a Cell-SELEX protocol previously reported by Sefah et al. [18] with several modifications. First, as human sperm cells used for in vitro studies are typically obtained from whole seminal fluid, rather than via traditional culture techniques, pre-selection cell growth and preparation methods were substituted with a density gradient centrifugation–based sperm cell isolation process [20]. Microscopic examination of cells pre- and post-isolation confirmed the successful separation of spermatozoa from other seminal leukocyte and epithelial cells (Fig. 1). Such isolation is vital to ensuring that aptamers are not selected against non-target cell types or other whole fluid components.

A randomized N40 ssDNA library containing fixed flanking regions was incubated with a defined number of sperm cells (which were progressively decreased between SELEX cycles 1–5 from 5 × 10^6^ to 1 × 10^6^ cells and kept at 1 × 10^6^ cells for all subsequent incubations) at the start of each round of selection. Aptamers able to bind to sperm targets were then recovered through centrifugation and eluted from the surface of cells before undergoing PCR amplification (to increase relative representation in subsequent selection rounds).

**Fig. 2:**
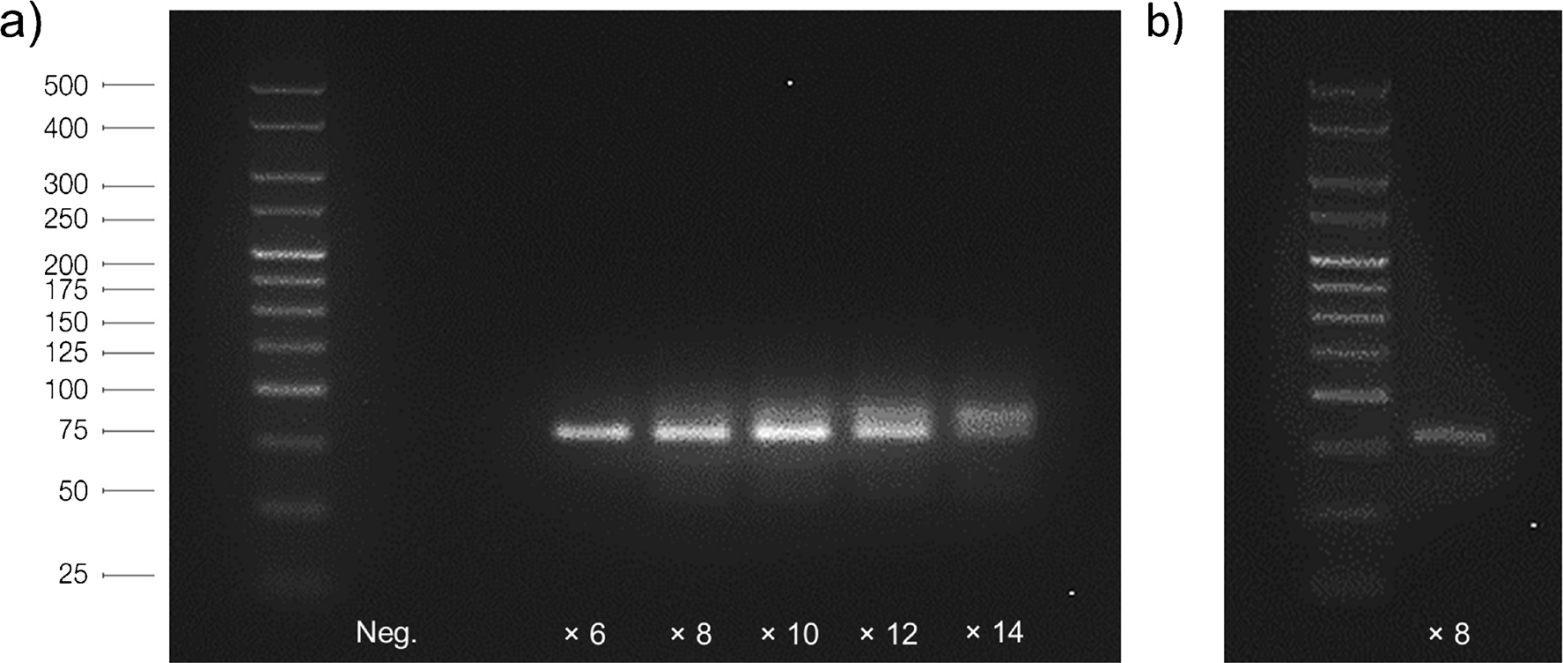
Gel electrophoresis images of **a** trial and **b** preparative PCR amplifications from the 4th round of the sperm cell aptamer selection process. Lane legends represent the number of amplification cycles performed or negative (Neg.) control samples

Trial PCRs were first conducted to determine the number of amplification cycles needed to generate the amounts of DNA required for further aptamer-cell incubations. Amplified products were successfully detected across all selection cycles using agarose gel electrophoresis. These products were found to run at approximately the same speed as the 75-bp fragment of a utilized Hyperladder DNA standard, which is consistent with the theoretical size (76 bp plus additional 6-FAM and biotin functionalisation) of dsDNA sequences (Fig. 2a).

One interesting effect observed during trial PCR analysis was the presence of a secondary DNA band running slightly slower than identified dsDNA products in samples amplified over a certain number of cycles. This effect varied between each selection round and was not found to be specific to a set number of amplification cycles. Previous studies have attributed such effects to the formation of PCR by-products as a result of the repetitive amplification of randomized ssDNA libraries [22]. These by-products are likely to originate from the hybridization of ssDNA sequences to complementary bases within the random N40 region, which then serve as PCR primers. Extension of sequences at these priming regions by DNA polymerases then results in the formation of longer dsDNA products, which run at a marginally reduced speed on electrophoretic gels. Consequently, optimal PCR cycle numbers used for subsequent preparative amplifications were chosen based on the trial PCR samples where only single product bands were observed (Fig. 2b).

Double-stranded amplification products were then subjected to strand separation using a Pierce NeutrAvidin agarose resin and NaOH denaturation to recover sense ssDNA sequences. After desalting, aptamer pools were quantified before being diluted for input into subsequent SELEX round cell incubations. A general increase in the concentration of recovered ssDNA during each round of selection was observed across up until round 11 of the Cell-SELEX process. This increase may indicate the success of aptamer selection, with greater target-sequence binding resulting in increased amounts of eluted ssDNA template available for amplification (which in turn results in higher PCR product concentrations).

### Massively parallel sequencing and data analysis

Another  modification made to the employed Cell-SELEX protocol [18] included the use of MPS technologies to elucidate the sequences of potential binding ligands. Such technologies were chosen for use in this study to elicit a greater amount of sequence information from enriched aptamer pools, as well as to avoid the time-intensive cloning and colony-picking methods associated with traditional Sanger sequencing. Several research groups have already shown the effectiveness of MPS analysis within aptamer selection experiments to allow for the detection of ligands at earlier rounds of selection and greater characterization of the evolution of enriched pools between SELEX cycles [23–28].

Three separate instances of high-throughput sequencing were conducted using the Illumina Miseq platform to determine the sequence and relative abundance of ssDNAs contained within early (rounds 1–3), mid (rounds 8–10), and late (round 14) SELEX cycle aptamer pools. Significant data processing was required to isolate random N40 bases from fixed flanking regions, as well as to remove any undesirable selection and/or sequencing artefacts. Such artefacts include sequences of incorrect length (originating as amplification by-products as already discussed) or those containing mismatched constant regions.

**Fig. 3:**
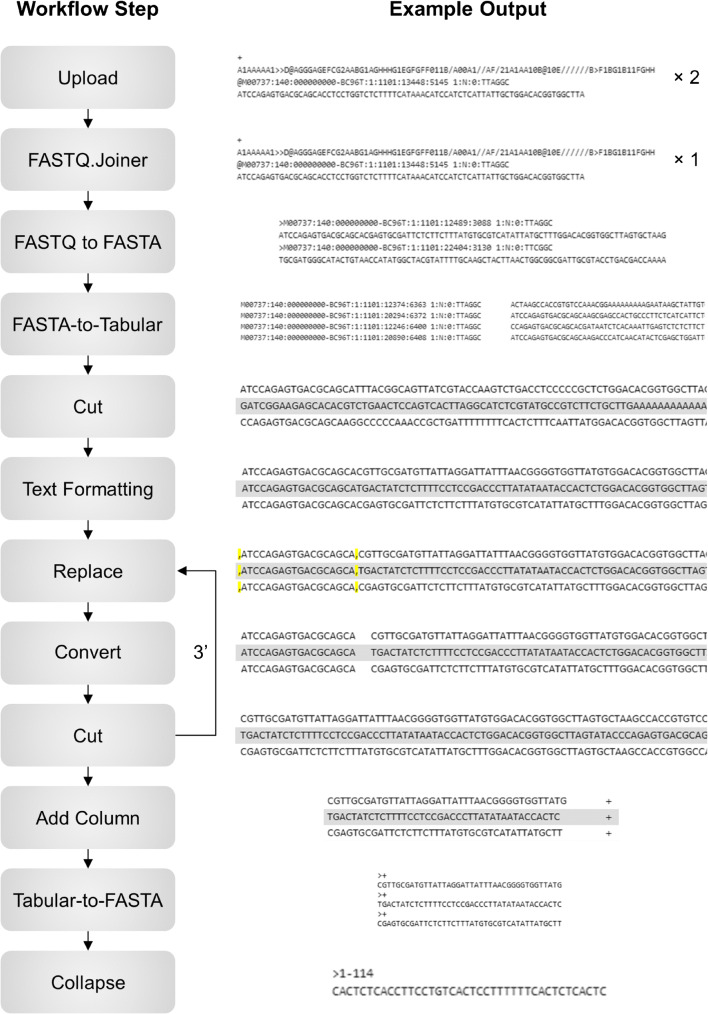
Scheme of the developed Cell-SELEX Galaxy pipeline and example data outputs

This was achieved using Galaxy, an online bioinformatics management system that allows the construction of custom data processing workflows for the analysis of complex genomics datasets [29]. A Cell-SELEX workflow (made available at https://usegalaxy.org/u/jamesgooch/w/cell-selex) for the processing of MPS data obtained from this study was developed according to methods reported by Thiel et al. [21]. This automated pipeline is able to provide collated sequence and read count information from a single input of generated raw forward and reverse-read FASTQ files without further user contribution (Fig. 3). The individual steps of this workflow (as separate Galaxy ‘tools’) were as follows:

1. Upload—FASTQ files from both forward and reverse sequences reads for a given round of selection are first uploaded to the Galaxy web server.
2. FASTQ.Joiner—Both forward and reverse paired-end sequence reads are merged together, allowing the simultaneous processing of datasets.
3. FASTQ to FASTA—Phred quality scores assigned to each nucleotide are removed by converting files to a FASTA format.
4. FASTA-to-Tabular—Files are then converted to a text-based format that allows for easier sequence manipulation.
5. Cut—The unique identifier assigned to each read is removed, leaving only raw sequence data.
6. Text Formatting—Sequences that do not meet specific text-based criteria are removed from the dataset. In this workflow, sequences are retained if both constant flanking regions, as well as a central series of nucleotides of 38–42 bases in length, are present. Longer amplification by-products are removed at this stage.
7. Replace—A comma character is placed at the end of 5′ constant flanking sequences, separating them from central N38-42 base regions.
8. Convert—Sequences are split based on the position of inserted comma values, placing 5′ constant regions into a separate column.
9. Cut—Columns containing 5′ constant region sequences are subsequently removed from the dataset.
10. Replace, convert, cut—Steps 7–9 of the workflow are repeated in order to remove 3′ flanking regions, which are separated by placing a comma character at the beginning of the constant sequence. After this stage, only N38-42 sequences are present within the dataset.
11. Add Column—An additional column is added to the dataset containing arbitrary ‘+’ character values against each sequence. These values act as sequence identifiers, allowing file conversion to a FASTA format.
12. Tabular-to-FASTA—Files are converted back from tabular to FASTA file formats, allowing for the sequences to be collapsed.
13. Collapse—Repeated identical sequences are reduced into a single read and assigned a count value corresponding to the number of duplicated sequences present within a sample.

Whilst application of the developed Galaxy data processing workflow resulted in a general decrease in the total number of sequence reads in all MPS datasets, this reduction was found to be less substantial for the sample obtained from the final round of selection. Such results may potentially be taken as an indication of successful selection, with increased enrichment of viable aptamer sequences (that meet data processing parameters) compared to amplification of sequencing artefacts.

Despite the success of other research groups in using MPS to identify viable aptamer candidates in less than 10 rounds of selection, analysed samples obtained from early (rounds 1–3) and mid (rounds 8–10) Cell-SELEX cycles did not contain any sequences that were repeated more than twice (after data processing). However, a total of 13,138 sequences were found to be repeated multiple times in the MPS dataset obtained from the final round of selection (round 14). In fact, 15 sequences were found to possess read counts greater than 25 (Table 4), thereby demonstrating potential for the recognition of human sperm cells.

**Table 4:** Sequences obtained from round 14 of selection exhibiting more than 25 total reads

Sequence ID	Read count	Length (bp)
SP1	252	40
SP2	61	40
SP3	53	40
SP4	48	39
SP5	41	40
SP6	37	40
SP7	37	40
SP8	37	40
SP9	33	40
SP10	30	40
SP11	29	40
SP12	28	40
SP13	28	39
SP14	28	40
SP15	26	40

### Microscale Thermophoresis

To confirm the binding of generated ligands to sperm cells, sequences SP1, SP2, and SP3 (the three candidates displaying the highest read counts from round 14 aptamer pool sequencing) were synthesized with 5′ 6-FAM fluorophores to enable their use within MST interaction experiments. MST, or ‘thermo-optical measurement’, is a recently reported technique that exploits differences in the physical movements of individual molecules passing through a spatial temperature gradient [30]. This phenomenon, known as ‘thermophoresis’, is largely dependent on the size, charge, and hydration shell properties of a molecule, which can all be altered as a result of a binding event (or associated conformational changes induced within a binding ligand). MST analysis can therefore be used to obtain accurate binding information from a sample by monitoring the concentration of fluorescently labelled molecules present within a defined region of elevated temperature, and how this concentration changes as a result of ligand-target interaction [31]. MST analysis was chosen for use within this study due its ability to characterize binding events in free solution, thereby avoiding the need for aptamer immobilization (which can alter binding ability and decrease the accuracy of interaction measurements).

**Fig. 4:**
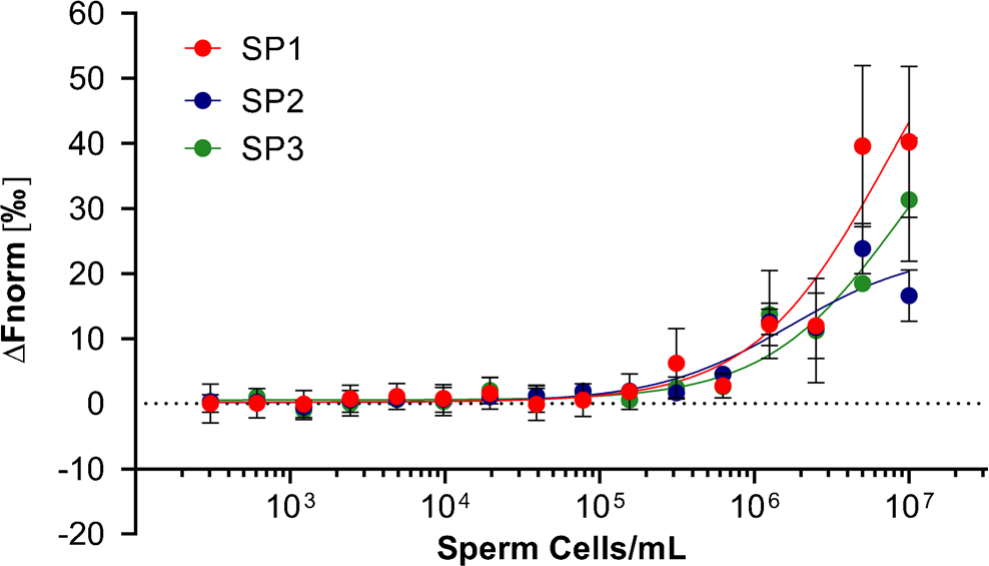
MST dose-response curves for aptamer candidates SP1, SP2, and SP3 against serially diluted sperm cell suspensions. Error bars = s.d.; *n* = 3 (independent experiments)

Figure 4 shows normalized fluorescence changes for sequences SP1, SP2, and SP3 as a function of sperm cell concentration. With increased emission signals observed in response to rising cell concentrations, these results successfully demonstrate the binding of each aptamer candidate to its intended target. It should be noted that whilst sequence SP1 displayed the highest MST response amplitude towards sperm cells during binding experiments, sequence SP3 possessed the highest average signal-to-noise ratio, which is often a better indicator of the quality of binding data [32].

In the ‘WHO laboratory manual for the examination and processing of human semen’, the reference range given for the concentration of sperm cells in the ejaculate of a healthy individual is 15–213 × 10^6^/mL (5th and 95th centiles, respectively) [20]. With positive increases in MST emission signals observed during the incubation of each aptamer candidate with every sperm sample above a concentration of 10 × 10^6^/mL, confidence may be given in the ability of these aptamers to bind sperm cells within neat semen samples obtained as part of sexual assault investigations. Additionally, whilst there is no ‘typical’ volume of semen recovered during such investigations, the total volume of sperm cell samples used during MST analysis was 5 μL, which would typically be considered a ‘trace’ amount within forensic casework. It is also important to note that no fluorescence changes were observed during the incubation of all sperm cell samples with sequences SP1, SP2, and SP3 containing constant region sequences.

It should be noted that identical MST experiments were also carried out using aptamers SP1, SP2, and SP3 synthesized to include the constant region sequences utilized as part of the Cell-SELEX process in order to examine potential effects on ligand-target binding. No fluorescence changes were observed during the incubation of each of these sequences with any sperm cell sample (subsequently preventing the plotting of MST dose-response curves), thereby demonstrating the selectivity of interactions between sperm cells and original SP1, SP2, and SP3 sequences not containing additional constant regions.

One limitation associated with the use of MST analysis for assessing the binding of fluorescent aptamers to whole-cells is the inability to determine accurate equilibrium dissociation constant (*K*_D_) values as a measure of binding affinity. This is due to the fact that it is not possible to determine the exact molar concentration of a target in a population of whole sperm cells without radiolabelled aptamers [33, 34]. Previous studies have attempted to overcome this issue by assigning arbitrary molarity values to cell populations based on estimated potential concentration ranges [35]. However, the authors of these studies have also questioned the reproducibility of this approach, given the variability of antigen expression between cell batches [35]. A more suitable solution may be to conduct MST experiments in which consistent concentrations of fluorescently labelled sperm cells are incubated with a serial dilution of aptamers at known molarities, which could then be used to calculate *K*_D_ values. However, for this to occur, a method for the reliable labelling of sperm cells with a consistent number of fluorophores (and in a way that does not inhibit aptamer binding) would first need to be identified. Despite these limitations, MST analysis using fluorescently labelled aptamers may still be considered a useful method for providing a robust indication of successful aptamer-cell binding.

**Fig. 5:**
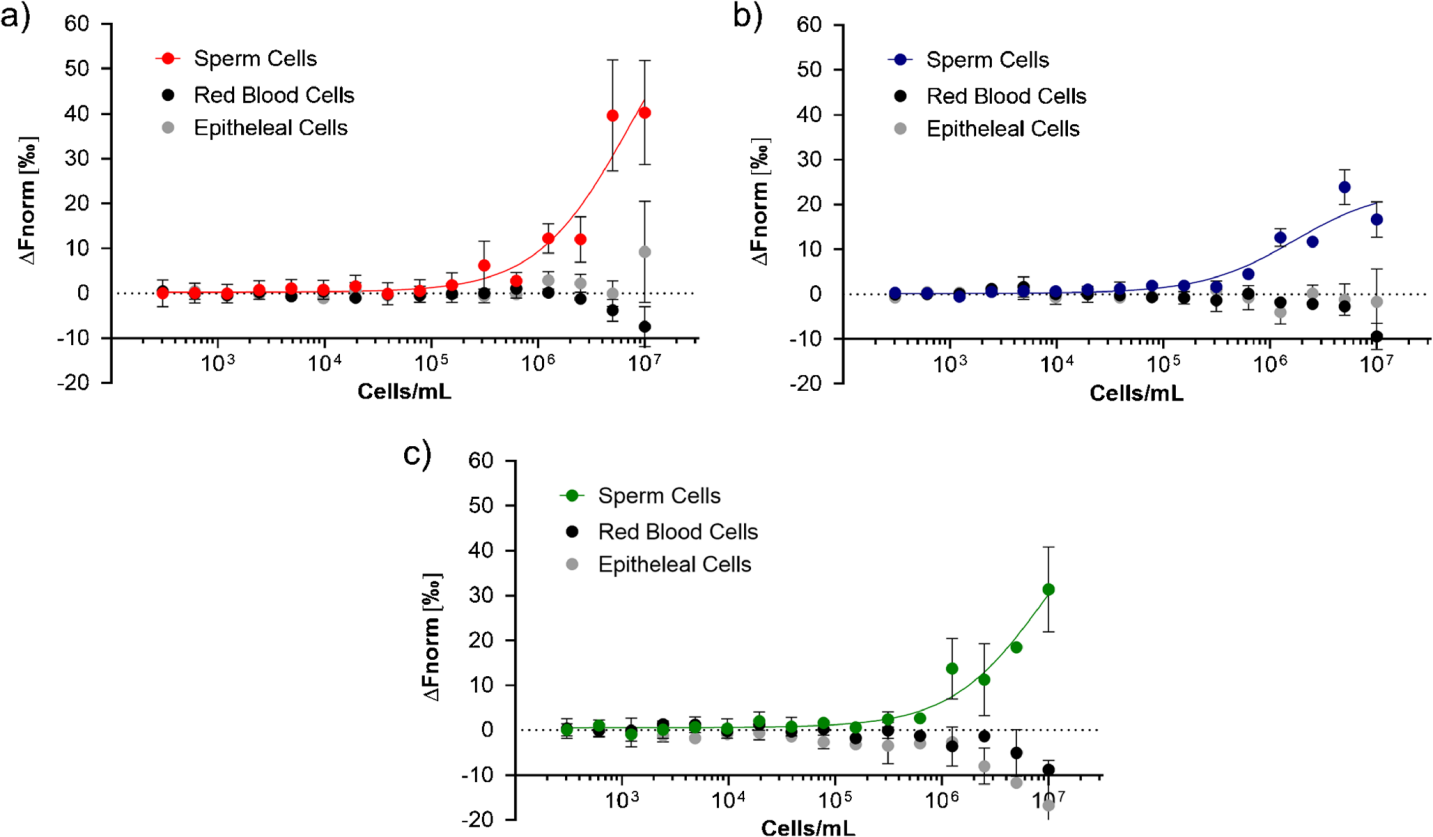
MST dose-response curves for aptamer candidates: **a** SP1; **b** SP2; and; **c** SP3 against serially diluted sperm cell, red blood cell, and epithelial cell suspensions. Error bars = s.d.; *n* = 3 (independent experiments)

MST experiments were also undertaken to assess the potential for the non-specific binding of sequences SP1, SP2, and SP3 to non-target cell types. For these experiments, human red blood cells and epithelial cells collected from human saliva were chosen as negative controls for both their relevance to forensic analysis and their ability to be isolated from biological fluids, rather than prepared through cell culturing techniques. These targets were tested against aptamer candidates in the same manner as sperm cells. In all cases, no interaction was observed between aptamers and negative control cells (Fig. 5). Such results demonstrate excellent selectively of the selected aptamers increasing their potential for future use within biosensing assays and other analytical platforms.

### Enzyme-linked oligonucleotide assay

With previous studies demonstrating that proteins present on the surface of human sperm cells are not only expressed differently between individuals, but are also heterogeneously expressed across cell populations obtained from the same individual [36], it is crucially important for any potential future application to forensic casework that the aptamer sequences reported here are capable of binding to sperm cells from different donors.

A series of additional binding experiments were therefore undertaken, in which sequences SP1, SP2, and SP3 were incubated with dilution series of sperm cells obtained from two additional healthy donors (3 donors total) in enzyme-linked oligonucleotide assays (ELONA). ELONA is a plate-based technique for the detection of aptamer-target complexes, in which a protein or whole-cell target is first absorbed to a solid surface and subsequently incubated with biotinylated aptamer(s). Streptavidin-conjugated horseradish peroxidase (SA-HRP) is then conjugated to captured aptamers and used to catalyse the reaction of a chromogenic substrate (the absorbance of which can be measured via UV-Vis spectroscopy) [37]. This assay format was specifically selected to demonstrate that interactions between sperm cells and generated aptamer sequences can also be successfully observed through binding characterization techniques other than MST.

Sperm cells at different dilutions (from 1:10 to 1:1000, starting at an initial stock concentration of 4.7 × 10^6^ cell/mL) were therefore first adsorbed to the wells of MaxiSorp™ flat-bottom 96-well plates for 1 h at 37 °C. Each well was then washed three times with wash buffer to remove unbound cells before the blocking of any remaining well surface through the addition of a blocking buffer containing 1% BSA. Constant concentrations of each biotinylated aptamer, along with a randomized control sequence (5′-biotin-ACTAAGCCACCGTGTCCA-3′), were then incubated with sperm dilution samples (and with negative wells containing no cells) for 1 h at room temperature. All wells were again washed three times with wash buffer to remove unbound aptamers. Next, SA-HRP was added to each sample for a period of 45 min to allow conjugation to any biotinylated aptamers present on the surface of adsorbed sperm cells. Any unbound enzyme was then removed by a further three washes. Lastly, TMB substrate solution was added to each well to react with HRP, forming a colorimetric product, which after acidification with sulphuric acid, allowed the observation of aptamer-target binding through measuring sample absorbance at 450 nm.

**Fig. 6:**
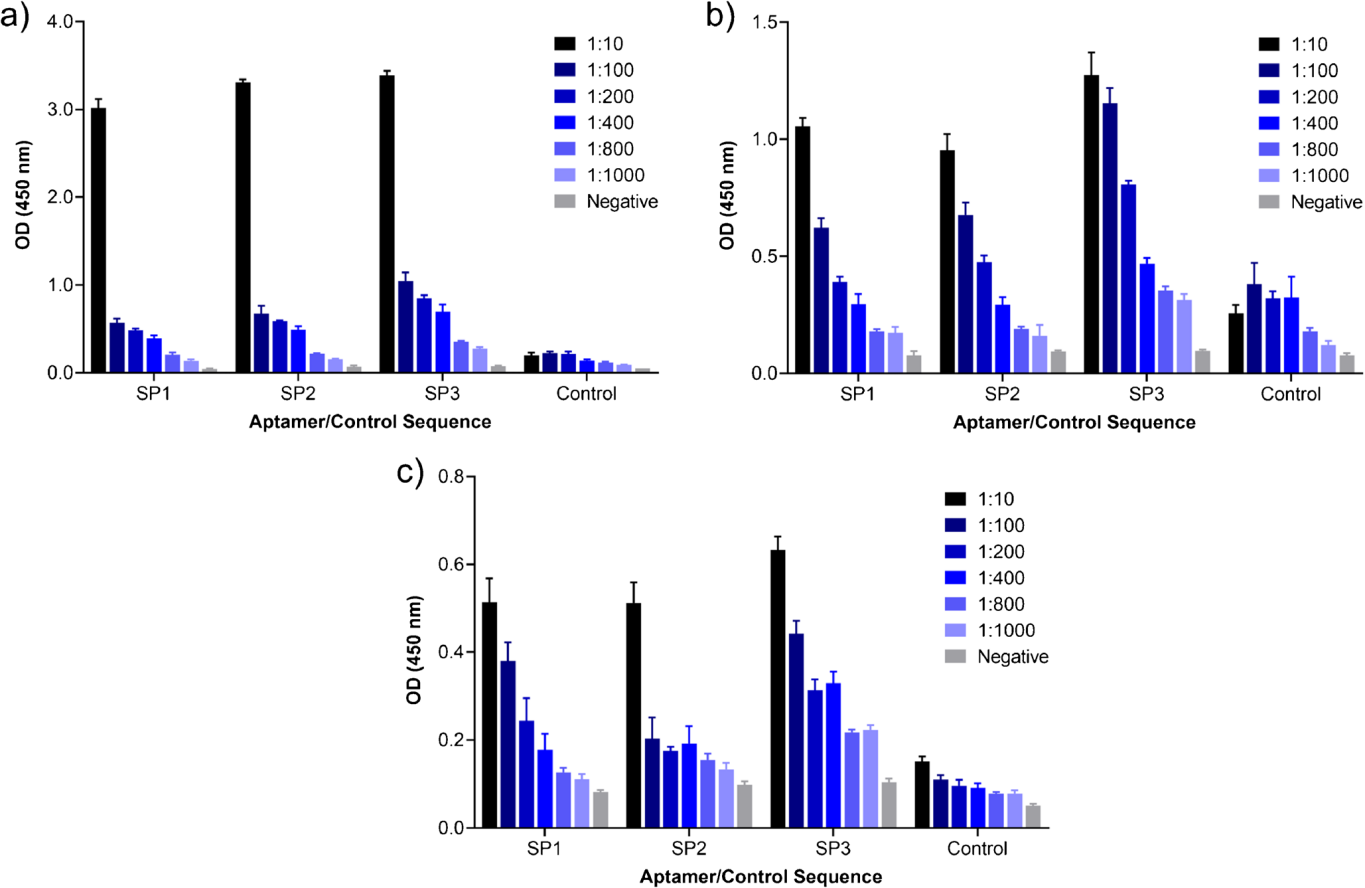
ELONA absorbance responses observed as a result of interactions between aptamers SP1, SP2, and SP3 (compared to a randomized control sequence) and sperm cell dilutions obtained from **a** donor 1; **b** donor 2; and **c** donor 3. Error bars = s.d.; *n* = 4 (independent experiments)

Figure 6 shows ELONA absorbance values obtained following the incubation of aptamers SP1, SP2, and SP3 (as well as a control sequence) with dilutions of sperm cells obtained from three separate donors. For each donor/aptamer combination, consistent increases in absorbance were observed with greater sperm cell concentrations, indicating dose-dependent binding responses and demonstrating the ability of each aptamer to interact with sperm cells from different individuals. In general, sequence SP3 produced the greatest substrate response across all donors, which is consistent with data from MST characterization experiments, where the same sequence produced the greatest signal-to-noise values. It is also important to note that substrate absorbance responses observed upon the incubation of the randomized control sequence with sperm cell dilution samples from all donors were low in comparison to those produced by SP 1–3, thereby demonstrating that sperm cell binding is largely achieved as a result of the specific sequences of identified aptamers (and is not due to non-specific ssDNA-cell interactions).

## Conclusions

A number of promising ssDNA aptamer candidates have been raised towards the recognition of human sperm cells using in vitro Cell-SELEX and massively parallel sequencing methods. A total of 14 rounds of selection were performed following a previously reported Cell-SELEX protocol, which was modified to include the isolation of sperm cells from human seminal fluid.

MPS of enriched aptamer pools was also used to monitor selection progress between rounds over traditional cloning and Sanger sequencing methods. MPS analysis was chosen for inclusion in this work for its ability to generate greater sequence information from enriched aptamer pools, with the additional possibility of identifying promising aptamer candidates within a reduced number of selection rounds (although this did not actually occur within this particular study).

A novel data processing pipeline was constructed using the online informatics workflow management system Galaxy, for elucidating the sequence structure of potential binding ligands. Whilst specifically developed for the analysis of data obtained within this study, this pipeline may have potential use in future SELEX experiments (towards both cellular and non-cellular targets) employing a similar N40 library template (although any library may be used through minor alterations to the text files used to identify constant region sequences and by defining an appropriate N region length).

Although these sperm cell–specific sequences were primarily raised for use within forensic analysis, either as part of cell identification assays or through the incorporation of sequences into solid-phase aptamer-functionalised materials (AFMs) for the capture and isolation of spermatozoa from vaginal epithelial cells, it is envisioned that such ligands may also have application to other areas of biomedical testing, including fertility evaluation.

To the best of our knowledge, this study also represents the very first use of MST to characterize binding interactions between aptamers and whole cells. MST experiments conducted in this work successfully established the ability of generated aptamer candidates to selectively bind to sperm cells over a series of non-target cells, whilst further ELONA experiments also demonstrated binding to cells obtained from different donors (in comparison to a randomized negative control sequence). However, further studies are needed to determine the specific cell surface antigens involved in aptamer binding, before accurate *K*_D_ values can be obtained for these sequences.

## Data Availability

The datasets generated during and/or analysed during the current study are available from the corresponding author on reasonable request.
